# Role of CD44 in Chemotherapy Treatment Outcome: A Scoping Review of Clinical Studies

**DOI:** 10.3390/ijms25063141

**Published:** 2024-03-08

**Authors:** Zihao Wu, Jillian Lu, Andrew Loo, Nathan Ho, Danny Nguyen, Po Yueh Cheng, Ali I. Mohammed, Nicola Cirillo

**Affiliations:** 1 Melbourne Dental School, The University of Melbourne, 720 Swanston Street, Carlton, VIC 3053, Australia; zwwu4@student.unimelb.edu.au (Z.W.); mohammed.a@unimelb.edu.au (A.I.M.); 2 School of Dentistry, University of Jordan, Amman 11942, Jordan

**Keywords:** CD44, hyaluronic acid, chemotherapy, chemoresistance, treatment outcome, clinical outcome

## Abstract

Cluster of differentiation 44 (CD44), a cell surface adhesion molecule overexpressed in cancer stem cells, has been implicated in chemoresistance. This scoping review, following PRISMA-ScR guidelines, systematically identified and evaluated clinical studies on the impact of CD44 expression on chemotherapy treatment outcomes across various cancer types. The search encompassed PubMed (1985–2023) and SCOPUS (1936–2023) databases, yielding a total of 12,659 articles, of which 40 met the inclusion criteria and were included in the qualitative synthesis using a predefined data extraction table. Data collected included the cancer type, sample size, interventions, control, treatment outcome, study type, expression of CD44 variants and isoforms, and effect of CD44 on chemotherapy outcome. Most of the studies demonstrated an association between increased CD44 expression and negative chemotherapeutic outcomes such as shorter overall survival, increased tumor recurrence, and resistance to chemotherapy, indicating a potential role of CD44 upregulation in chemoresistance in cancer patients. However, a subset of studies also reported non-significant relationships or conflicting results. In summary, this scoping review highlighted the breadth of the available literature investigating the clinical association between CD44 and chemotherapeutic outcomes. Further research is required to elucidate this relationship to aid clinicians in managing CD44-positive cancer patients.

## 1. Introduction

Chemotherapy is one of the principal methods of cancer treatment [1]. Effective chemotherapeutic treatments improve the survival rate and quality of life of cancer patients. Yet challenges persist, with some tumors displaying intrinsic resistance to chemotherapy and others developing resistance after an initial positive response [2].

Cluster of differentiation 44 (CD44) is a cell surface glycoprotein expressed in a large number of cell types [3] with more than 20 isoforms [4]. Among its more prominent isoforms are standard CD44 (CD44s), as well as variant isoforms such as CD44v6 and CD44v9, each contributing to the diverse functions of CD44 in cellular processes [4]. It is a multi-functional cell surface adhesion receptor that binds primarily to hyaluronic acid (HA) [3]. HA, also known as hyaluronan or hyaluronate (salt), is a polysaccharide ubiquitous in the extracellular matrix [5]. HA–CD44 binding regulates transporter activities and activates signaling pathways that affect cell–matrix adhesion, cell migration, proliferation, differentiation, and survival [6]. These processes are vital for both normal cellular function and malignancy development. They are found in both healthy tissue and tumor tissue with varying expression levels [7].

CD44 is highly expressed in many cancers and is also recognized as a cancer stem cell (CSC) biomarker [8,9]. Its interaction with extracellular matrix ligands promotes cell migration and invasion, which are commonly involved in metastases. CD44 also plays a key role in regulating the properties of CSCs, including self-renewal, tumor initiation, and chemoresistance [10,11]. Notably, studies have highlighted a positive correlation between heightened CD44 levels and adverse prognostic outcomes in various cancers, including breast cancer, colorectal cancer, pancreatic carcinoma, and prostate cancer [12,13,14,15].

CD44 also interacts with other signaling receptors, such as transforming growth factor-β (TGF-β) and epidermal growth factor receptor (EGFR), influencing a variety of cancer signaling cascades (notably PI3 kinase-Akt and MAP kinases pathways) which ultimately lead to chemoresistance, invasion, cell proliferation, and survival [16]. Inhibition of CD44 has also been associated with enhanced chemosensitivity of cancer cells [17].

Investigations into chemotherapy drug efflux activity in human T-cell acute lymphoblastic leukemia (T-ALL) cell lines revealed a positive correlation between enhanced drug efflux activity and increased CD44 expression [18]. Furthermore, HA-CD44 interaction was found to induce ankyrin (a cytoskeletal protein) binding to multi-drug resistance 1 (MDR1), resulting in the efflux of chemotherapeutic drugs and the development of chemoresistance in human breast and ovarian cell lines [19]. Collectively, these findings suggest a potential role for CD44 in regulating drug efflux mechanisms, indicating its potential involvement in cellular resistance to chemotherapy.

While many in vitro and animal studies have shown that CD44 confers chemoresistance, the relationship between CD44 and chemotherapy treatment outcomes in cancer patients is ambiguous [20]. Hence, this scoping review aims to systematically review the current clinical literature to elucidate the impact of CD44 on chemotherapy treatment outcomes in cancer patients, addressing the existing uncertainties in this vital area of cancer research.

## 2. Results

### 2.1. Search Results

**Figure 1:**
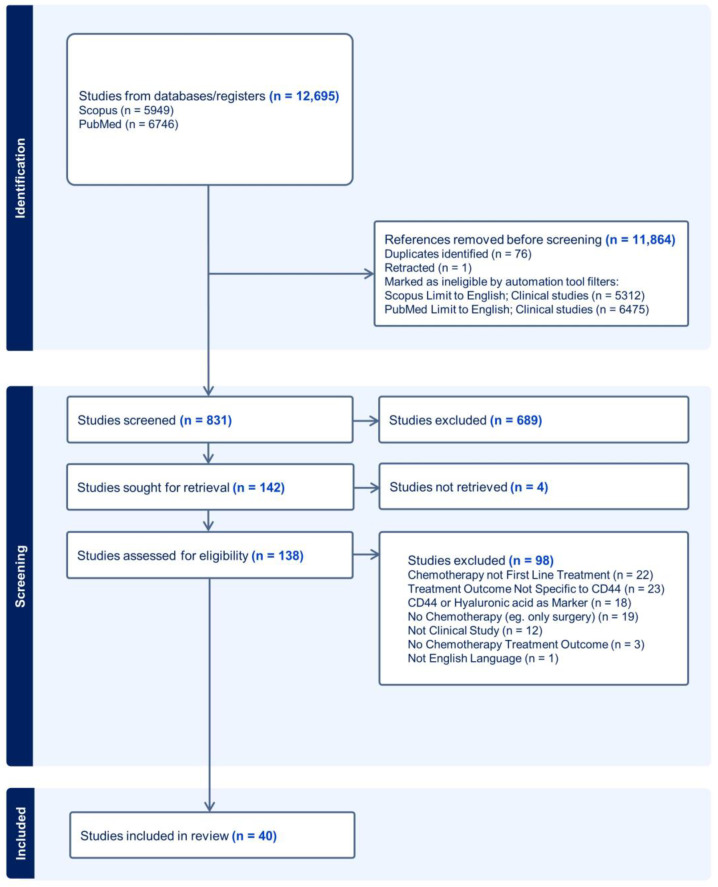
PRISMA-ScR diagram of the article selection process (last search in June 2023).

The search conducted on Scopus and PubMed databases yielded a total of 12,659 articles. Automated exclusion filters within Scopus and PubMed were used to limit these results to English and clinical studies, thus removing non-English, non-human, and review articles. Out of the remaining 908 articles, 76 duplicates and 1 retracted article [21] were removed using Endnote. The titles and abstracts of the remaining 831 publications were screened in accordance with the eligibility criteria. A total of 142 articles met the inclusion criteria and were sought for retrieval. A total of 4 articles were unable to be retrieved, and the remaining 138 articles were screened for full-text eligibility. As many as 98 studies were excluded according to the eligibility criteria, and the remaining 40 studies were included in the qualitative synthesis. The selection process is summarized in Figure 1. Studies were reported according to the updated PRISMA-ScR guidelines. The kappa scores for both rounds of screenings are presented in Supplementary Table S1. The kappa scores range from 0.7 to 0.91, demonstrating substantial to almost perfect agreement.

After full text screening, data from the list of selected articles were presented as an extraction table in the Supplementary Table S2. This included the cancer type, sample size, interventions, control, treatment outcome, study type, detailed interventions, CD44 variants and isoforms, CD44 quantification, major findings, the effect of CD44 on chemotherapy outcome (e.g., overall survival, tumor recurrence, resistance to chemotherapy), and other cancer molecular biomarkers.

### 2.2. Effect of CD44 Expression on Chemotherapy Outcome

The results were categorized according to the effect of CD44 expression on chemotherapy treatment outcome. In summary, three studies reported a positive effect of increased CD44 expression on chemotherapy treatment outcome [22,23,24], thirteen studies reported no significant effect between CD44 expression and chemotherapy treatment outcome [25,26,27,28,29,30,31,32,33,34,35,36,37], and twenty-four studies reported a significant negative effect of increased CD44 expression on chemotherapy treatment outcome [38,39,40,41,42,43,44,45,46,47,48,49,50,51,52,53,54,55,56,57,58,59,60,61]. These studies are summarized in Table 1, Table 2 and Table 3, respectively.

### 2.3. Effect of CD44 Expression on Outcome of Various Interventions

Studies were further stratified based on the types of interventions. Nine studies involved chemotherapy as the only treatment [35,36,45,50,51,54,55,59,61], eight studies only involved radiochemotherapy [23,24,29,31,34,38,49,56], fifteen studies involved chemotherapy and surgery [25,26,27,30,33,39,41,42,44,46,47,52,53,57,58], and seven studies involved chemotherapy, radiotherapy, and surgery [22,28,37,39,43,48,60]. One study involved chemotherapy and hormonal therapy [40]. Table 4 reports the number of articles with negative, no, or positive associations between CD44 expression on chemotherapy treatment outcome stratified by different types of intervention. Note that chemotherapy is the first line of treatment in all the different types of intervention. In summary, CD44 expression consistently demonstrates a negative impact on chemotherapy outcomes across various interventions, particularly in combined treatments involving radiotherapy or surgery. While some interventions show no significant effect, limited evidence suggests a potential positive impact in some cases. However, it is essential to note variations in findings across different types of interventions and the absence of positive effects in several categories. The findings underscore the complex relationship between CD44 expression and chemotherapy response, highlighting potential variations based on the specific combination of treatment modalities.

The effect of CD44 expression on chemotherapy treatment outcome categorized by the most common chemotherapy drugs used in studies included in this review is reported in Table 5 (5-fluorouracil [28,31,32,33,35,37,38,41,43,46,47,48,60], cisplatin [23,29,30,33,34,38,39,46,49,58,61] and docetaxel [34,38,46,50,58,61]). Other drugs used include adriamycin/doxorubicin [30,46,50,56], cyclophosphamide [35,43,46,50,56], cetuximab [29,38,49], paclitaxel [22,35,59], epirubicin [39,43], carboplatin [22,38], and etoposide [39,45]. The following drugs are all mentioned by one article each: methotrexate [30], vinorelbine [34], leucovorin [37], bleomycin [39], idarubicin [45], ara-c [45], ifosfamide [30], thioguanine [45], amifostine [49], sunitinib [54], bevacizumab [55], anthracycline [57], taxane [57], capecitabine [58], temozolomide [24], trastuzumab [59], lapatinib [59], and capecitabine [58]. Elevent studies did not specify the chemotherapeutic drug or regime used to treat the patients [25,26,27,36,40,42,44,51,52,53]. Data analysis shows that CD44 overexpression tends to have a predominantly negative effect for both single and combination chemotherapy. Notably, for treatments using a single chemotherapeutic drug, only cisplatin and temozolomide showed positive effects of CD44 overexpression on chemotherapy outcomes.

### 2.4. Effect of CD44 Expression on Chemotherapy Treatment Outcome in Different Types of Cancer

The types of cancer in the studies included in this review are breast cancer [25,26,35,40,43,50,51,52,53,57,59], rectal cancer [28,31,32,37,47,48,60], head and neck cancer [29,38,49,51], cervical cancer [23,34,39], esophageal cancer [22,33,46], leukemia [36,45], osteosarcoma [27,30,44], colon cancer [41,55], kidney cancer [42,54], gastric cancer [58], glioblastoma [24], lung cancer [61], and lymphoma [56], as summarized in Table 6. Taken together, the results show that CD44 overexpression tends to have a predominantly negative impact on chemotherapy outcomes across various cancers, with a limited number of instances showing no effect or a potential positive effect, highlighting the complex and varied relationship between CD44 expression and treatment response in different cancer types. These findings underscore the need for tailored approaches in understanding the impact of CD44 expression across different cancers.

## 3. Discussion

This scoping review systematically examined the relationship between CD44 expression and chemotherapy treatment outcomes across a diverse range of cancers. Most of the articles show an association between increased CD44 expression and poor chemotherapy treatment outcome (Table 3), aligning with findings from laboratory and animal studies that implicate CD44 in chemoresistance [62,63]. However, some studies reported that CD44 overexpression has no significant effect or even a positive association with treatment outcome (Table 1 and Table 2), highlighting the complexity of this relationship. Furthermore, we could not ascertain the possible role of concurrent administration of HA in cancer chemotherapy, although it is known that hyaluronan affects the synthesis of molecules involved in its own pathway, including CD44 [64]. All studies involved chemotherapy as the main first-line intervention, but combined interventions with other treatment modalities, such as chemoradiotherapy, radiotherapy, and surgical resection, were also included. Chemotherapy treatment outcomes were commonly measured through survival rates, recurrence rates, and response to therapy.

### 3.1. Relationship between CD44 and Chemoresistance

Twenty-Four studies report an association between CD44 and chemotherapy treatment outcome, leading to poor prognosis, reduction in overall survival, and increased recurrence of disease [38,39,40,41,42,43,44,45,46,47,48,49,50,51,52,53,54,55,56,57,58,59]. The association in these clinical studies is consistent with the findings in many laboratory and animal studies, showing that CD44 contributes to tumorigenicity, cell division, anti-apoptosis, metastasis, and chemoresistance [65,66].

From a mechanistic point of view, CD44 has been shown to mediate multiple cancer signaling pathways. CD44v6 activation leads to ezrin–radixin–moesin (ERM) activation, which promotes Ras activation [67], causing cell division and proliferation. It also acts as a co-receptor for receptor tyrosine kinase c-Met by binding and sequestering its ligand, HGF, leading to c-Met activation, which, in turn, causes VEGFR-2 and ERK activation in endothelial cells, ultimately contributing to angiogenesis [68]. CD44 also regulates matrix metalloproteinases (MMPs) expression through its downstream transcriptional factor target, Snail1. It promotes Snail1 translocation into the nucleus, upregulating the transcription and translation of MMPs which increase breast cancer cell invasion [65]. Furthermore, activated CD44 can induce hypoxia-inducible factor HIF1α binding to nuclear DNA to increase glycolysis, causing a metabolic shift in cells [69]. For chemoresistance, the interaction of CD44 with p300 and SIRT1 is found to regulate β-catenin signaling and NFκB-specific transcription activity, leading to MDR1 and Bcl-xL gene expression and chemoresistance in breast tumor [70]. CD44-positive prostate cancer cells were also reported to be resistant to docetaxel after a few months of treatment due to the elevation of AKT-dependent drug transporter ABCB1 and expression of class III β-tubulin [71]. The addition of hyaluronidase, an enzyme that hydrolyzes HA, sensitizes T-ALL cells to doxorubicin, suggesting that the chemoprotective effect of CD44 may require activation by HA ligand [18]. Clearly, CD44 plays a key role in tumorigenicity and chemoresistance via multiple cell signaling pathways, and this is in agreement with our results showing that more than half of the clinical studies in this review indicate that CD44-positive cancer patients have poor chemotherapy treatment outcomes.

Table 4, Table 5 and Table 6 summarize the effect of CD44 on chemotherapy treatment outcomes by stratifying the clinical studies into different interventions, drug types, and cancer types respectively. The rationale is to assess if some types of intervention, drug, or cancer are more associated with poor chemotherapy outcomes than other types.

For types of intervention, the negative effect of CD44 on chemotherapy treatment outcome is the highest in interventions with chemotherapy alone (80% of the studies) and the lowest in chemotherapy + radiotherapy (42% of the studies). This further demonstrates CD44’s role in chemoresistance. The relatively low negative effect for chemotherapy + radiotherapy intervention might be attributed to radiotherapy as there is no evidence that CD44 confers radioresistance.

For types of chemotherapy drug and cancer, both Table 5 and Table 6 indicated a trend wherein CD44 is associated with negative chemotherapy treatment outcome. Notably, for cancer type, the negative effect of CD44 on chemotherapy treatment outcomes is the highest in breast and head and neck cancer (72% and 75% of the studies). However, the number of clinical studies for each type of cancer is small, which limits the interpretation of the results.

### 3.2. Contradicting Studies

Although the majority of the studies obtained found that CD44 expression was associated with poorer chemotherapeutic outcomes, various studies had contradictory findings. Three studies found CD44 to be positively associated with treatment outcomes [22,23,24]. All three studies involved the use of chemoradiation as the treatment regime. The addition of radiotherapy may have contributed to the positive treatment outcome as CD44 is not associated with radioresistance.

Thirteen studies found that CD44 expression had no effect on treatment outcome [25,26,27,28,29,30,31,32,33,34,35,36,37]. These contradictory findings are likely attributed to individual variation amongst the different studies in terms of populations, interventions, and treatment outcome measures. Across the 13 studies with contradictory findings, treatment was performed on nine different cancer tissues. CD44 expression varies amongst different tissues, with certain isoforms being the predominant form in different cancers such as CD44v3 in head and neck squamous cell carcinomas [65]. Different isoforms are involved in different signaling pathways and would, therefore, have different clinicopathological effects affecting treatment outcomes [65].

Variability in the chemotherapeutic treatments administered may also contribute to inconsistent findings. Different studies use different drug dosages, different numbers of chemotherapy cycles, and different combinations of chemotherapy drugs. A common drug used in the studies was 5-fluorouracil, and it is known to produce better drug response rates when used in conjunction with other therapeutic agents [72]. All these heterogeneities may contribute to the contradicting studies.

Differences in treatment outcome measurements may also explain some of the contradictory findings. Measures such as pathological response or tumor regression look at the efficacy of treatment in the short term, as opposed to patient survival, which looks at efficacy over a prolonged period to provide prognostic data. Although all the studies measure patient survival post-treatment, each of them has its own specific criteria, which would result in varied findings and outcomes between studies. These differences may contribute to discrepancies in the findings.

### 3.3. CD44 and Hyaluronic Acid

This scoping review, while shedding light on the existing literature, underscores the imperative for additional research to elucidate the CD44–HA relationship from a clinical perspective. Despite including HA in the search strategy, the current evidence remains insufficient to comprehensively capture the nuanced clinical implications of CD44–HA interactions. While the influence of hyaluronic acid (HA) on cell signalling through its binding to CD44 is acknowledged [6], there is a notable absence of clinical studies thoroughly investigating the intricacies of the association between CD44 and HA and its consequential impact on chemotherapy treatment resistance across diverse tumor types. As recent studies have shown the clinical effectiveness of topical HA formulations in the reduction or prevention of mucositis in cancer patients undergoing chemotherapy [73,74], it is important to better understand the systemic and tumour-specific effect of the CD44–HA axis in clinical settings. Further research is imperative to elucidate this relationship from a clinical perspective before systemic HA medications can be safely used.

### 3.4. Limitations

There are several limitations in this scoping review in terms of internal and external validity.

For internal validity, the use of a specific search string across two different databases may have excluded relevant articles in other databases such as Ovid and Web of Science. Additionally, Cohen’s Kappa score of 0.70 for the title and abstract screening in Part 2 was lower than expected, indicating slight disagreement between reviewers with regards to the inclusion and exclusion criteria, which may also have led to the exclusion of relevant articles. Given the vast number of articles available and the heterogeneity of data, the risk of bias and the quality of the studies were also not assessed.

For external validity, this review summarized clinical articles with different target populations, interventions, drugs, and CD44 isoforms. The studies used different drugs and treatment regimens for different patients; hence, it is difficult to compare the effect of CD44 expression on treatment outcome across different interventions. Despite chemotherapy being the first-line treatment in all studies, other treatments such as radiotherapy and surgery make it difficult to isolate the association between CD44 expression and chemoresistance. Other limitations include different treatment outcome measures, a lack of control groups, and small sample sizes in the studies. For example, the study by Baek et al. [26] focused on serum CD44, whereas others assessed the histochemical expression of CD44 in tumor tissue. Some caution must be taken when interpreting results comparing different outcome measurements, such as immunohistochemical CD44 expression, serum CD44 levels, or serum HA levels. In addition, some studies also used different CD44 isoforms [27,39,42,57], which may have different effects on chemotherapy treatment outcomes. While attempts to identify contradictory findings amongst studies were made, differences in study design and treatment outcome measures make it difficult to reach a singular and definitive conclusion on the effect of CD44 on chemotherapy outcome.

However, as scoping review primarily examines the breadth rather than the depth of the evidence available, these limitations are consistent with what was initially expected, and future studies that seek to address such limitations will be beneficial.

In conclusion, while the majority of the evidence suggests a negative association between CD44 expression and chemotherapy outcomes, the complexity of cancer heterogeneity, treatment variations, and diverse outcome measures warrant cautious interpretation. Future research addressing these limitations will contribute to a more nuanced understanding of CD44’s role in chemotherapy response.

### 3.5. Future Directions

Further research is needed to examine the effects of CD44 expression across a wider variety of cancer types and chemotherapy drugs to achieve a more comprehensive understanding of the role of CD44 in cancer treatment outcomes. As highlighted in Table 6, current studies exploring the role of CD44 across varying cancer types remain limited, with most existing articles being relevant to breast and rectal cancer. This highlights the need for more clinical studies to be conducted across a wider range of cancer types, including those not covered in this review. In addition, as different chemotherapy drugs may interact with CD44 in a unique manner, future research should investigate the role of CD44 expression in response to different chemotherapy agents. The specific pathways in which CD44 induces chemoresistance should also be explored through the implementation of mechanistic studies. Furthermore, CD44 has various isoforms, and it would be interesting to investigate the effect of each isoform on chemoresistance and the clinical outcome in various cancers. In summary, CD44 expression and its role in chemotherapeutic outcomes warrants further investigation to bridge the gap in the current literature. This is clinically relevant as it impacts the chemotherapy options available and may have implications for whether combination therapies targeting CD44 should be used as adjuncts to conventional chemotherapy.

## 4. Materials and Methods

### 4.1. Search Strategy

This scoping review followed the updated preferred reporting items for systematic reviews and meta-analyses extension for scoping reviews (PRISMA-ScR) guidelines [75]. Since the aim of this scoping review was to summarize the effect of CD44 on chemotherapy treatment outcome in cancer patients, the search strategy involved the following three aspects:

1. Cancer patient;
2. CD44 or HA;
3. Chemotherapy treatment outcome.

To ensure comprehensiveness, the search was performed in both PubMed (1985–2023) and SCOPUS (1936–2023) databases to find relevant publications using the following search string (last accessed in June 2023):

((hyaluro* OR hyaluronic) OR (CD44 OR CD44*)) AND ((“cancer therap*” OR “cancer therapy”) OR (“cancer treatment” OR “cancer treat*”) OR (chemotherapy OR chemotherap*)) AND ((cancer OR cancer*) OR (tumour OR tumour*) OR (tumor OR tumor*)).

Key search terms were truncated to account for various nomenclatures of the same word. For example, “hyaluro*” is used to account for hyaluronic acid, hyaluronan, and hyaluronate, and “CD44*” is used to account for all the different CD44 isoforms. HA was included in the search strategy as it is the ligand of CD44.

### 4.2. Eligibility Criteria

A list of inclusion and exclusion criteria were established to facilitate the screening process.

Publications were included if they met the following inclusion criteria:

1. Studies investigating the relationship between CD44 or the HA–CD44 axis and chemotherapy treatment outcomes in cancer patients;
2. Peer-reviewed full-text articles presenting primary data;
3. Articles in the English language;
4. Clinical studies, including all phases of clinical trials, randomized controlled trials, comparative studies, and pragmatic studies.

Publications with any of the following exclusion criteria are excluded:

1. Not related to CD44 or HA on cancer;
2. Not specific to CD44 (even if HA is mentioned);
3. CD44 or HA used as biomarker;
4. No chemotherapy treatment outcome;
5. No chemotherapy treatment (e.g., only surgical treatment or radiotherapy);
6. Chemotherapy not first-line cancer treatment (e.g., surgery or radiotherapy prior to chemotherapy);
7. Not clinical study (e.g., in vitro, in vivo animal studies);
8. Articles with no primary data (e.g., review, meta-analysis);
9. Articles not in the English language.

It is important to note that even though this review aims to summarize the relationship between CD44 expression and chemotherapy treatment outcome, we included not only clinical studies with chemotherapy as the elective treatment but also studies with combination treatments (e.g., chemotherapy + radiotherapy, chemotherapy + surgery, and chemotherapy + radiotherapy + surgery) to gain a deeper understanding of the possible effects of CD44 in cancer treatment. It is noteworthy, however, that despite the inclusion of articles with combination treatments, chemotherapy must remain the first line of treatment in the selected studies.

### 4.3. Data Selection and Collection

After establishing the eligibility criteria, the search results underwent preliminary filtering using an automatic tool to limit articles to the English language and clinical studies.

The filtered search results were then divided into 3 parts for title and abstract screening. For each part, 2 reviewers independently evaluated the title and abstract based on the eligibility criteria to determine whether an article should be included. Disagreements between the 2 reviewers were resolved by a third-party judge. Cohen’s kappa was calculated for each part to evaluate inter-examiner reliability based on the following formula [76]:

$$k=\frac{p_{o}-p_{e}}{1-p_{e}},$$

where $p_{o}\;$ is the relative observed agreement among the 2 reviewers, and $p_{e}\;$ is the hypothetical probability of chance agreement. A kappa value of 0.80–0.90 indicates a strong level of agreement, and values above 0.90 indicate an almost perfect level of agreement [76]. Full-text screening followed the same process as title and abstract screening.

## 5. Conclusions

This scoping review highlights the role of CD44 in chemotherapy outcomes. Most of the results suggest that CD44 expression is related to poor chemotherapeutic outcomes and may be linked to chemoresistance. However, discrepancies in treatment outcomes were identified in the review, with several articles indicating that CD44 was associated with positive treatment outcomes or that it had no significant effect on outcomes. Further research is needed to examine the effects of CD44 expression across a wider variety of cancer types and chemotherapy drugs to achieve a more comprehensive understanding of the role of the CD44–HA axis in cancer treatment outcomes, paving the way for chemotherapy management of CD44-positive cancer patients.

## Figures and Tables

**Table 1:** Studies showing that CD44 expression has positive effects on chemotherapy treatment outcome.

Author, Year	Population	Intervention	Outcome/Effect Observed
Beukinga et al., 2021 [22]	Esophageal cancer (*n* = 43)	CRT (carboplatin and paclitaxel) + Surgery	CD44 associated with higher probability of achieving no residual cancer cells
Chopra et al., 2019 [23]	Cervical cancer (*n* = 148)	CRT (cisplatin)	Low levels of CD44 associated with locoregional relapse
Pinel et al., 2017 [24]	Glioblastoma (*n* = 122)	CRT (temozolomide)	CD44 associated with better progression-free survival

CRT = chemoradiotherapy.

**Table 2:** Studies showing that CD44 expression has no significant effect on chemotherapy treatment outcome.

Author, Year	Population	Intervention	Outcome/Effect Observed
Akay et al., 2022 [25]	Breast cancer (*n* = 91)	NACT + Surgery	No effect of C44 on survival or tumor regression
Baek et al., 2011 [26]	Breast cancer (*n* = 56)	NACT + Surgery	CD44 not associated with pathological complete response
Boldrini et al., 2010 [27]	Osteosarcoma (*n* = 52)	CT + Surgery	CD44 has no effect on survival rate
Deng et al., 2014 [28]	Rectal adenocarcinoma (*n* = 64)	NACT (5-FU) + RT + Surgery	CD44 not correlated to clinicopathological parameters
Grau et al., 2016 [29]	Head and neck SCC (*n* = 45)	CT (DDP or cetuximab) + RT	No significant differences in survival between CD44+ and CD44-
Hu et al., 2009 [30]	Osteosarcoma (*n* = 87)	CT (MTX + IFO + ADM + DDP) + Surgery	CD44 has no correlation to prognosis or differentiation
Kawamoto et al., 2012 [31]	Rectal cancer (*n* = 52)	CRT (5-FU + UFT)	No association between CD44 and clinical outcome or distant recurrence
Kojima et al., 2010 [32]	Rectal cancer (*n* = 102)	CRT (5-FU) + Surgery	No association between CD44 with overall survival or disease-free survival
Leone et al., 2016 [34]	Cervical SCC (*n* = 26)	NACT (NVB + DTX + IFO-NVB-DDP)/CRT (DDP)	CD44 not associated with worse outcome or treatment resistance
Minato et al., 2013 [33]	Esophageal SCC (*n* = 40)	NACT (5-FU + DDP) + Surgery	CD44 not related to pathological response rate
Tanei et al., 2009 [35]	Breast cancer (*n* = 108)	CT (PTX + 5-FU + epirubicin + CP)	CD44 is not associated with pathological complete response
Yokota et al., 1999 [36]	Leukaemia (*n* = 25)	CT	CD44 levels have no correlation to therapy response
Yoon et al., 2016 [37]	Rectal cancer (*n* = 145)	CRT (5-FU + leucovorin) + Surgery	No association between CD44 and recurrence-free survival or cancer specific survival

NACT = neoadjuvant chemotherapy; CT = chemotherapy; CRT = chemoradiotherapy; 5-FU = 5-fluorouracil; UFT = tegafur–uracil; RT = radiotherapy; SCC = squamous cell carcinoma; DDP = cisplatin; MTX = methotrexate; IFO = ifosfamide; ADM = adriamycin; NVB = vinorelbine; DTX = docetaxel; PTX = paclitaxel; CP = cyclophosphamide.

**Table 3:** Studies showing that CD44 expression has negative effects on chemotherapy treatment outcome.

Author, Year	Population	Intervention	Outcome/Effect Observed
Baschnagel et al., 2017 [38]	Head and neck SCC (*n* = 105)	CRT (DDP/carboplatin/cetuximab or DCF + DDP + 5-FU)	High CD44 predicts poor loco-regional control and prognosis
Costa et al., 2001 [39]	Cervical carcinoma (*n* = 21)	NACT (DDP + epirubicin + VP-16 + bleomycin) + Surgery + RT	Reduced CD44 associated with increased recurrence-free survival and overall survival
Elbaiomy et al., 2020 [40]	Breast cancer (*n* = 76)	CT + Hormonal therapy	High CD44 predicts poor response to treatment and shorter progression-free and overall survival
Gerger et al., 2011 [41]	Colon cancer (*n* = 234)	Adjuvant CT (5-FU-based) + Surgery	CD44 is associated with increased tumor recurrence
Ghanem et al., 2002 [42]	Nephroblastoma (*n* = 61)	NACT + Surgery	Increased expression of CD44 correlated with clinical progression and tumor-related death
Gong et al., 2010 [43]	Breast cancer (*n* = 192)	NACT (5-FU + epirubicin + cyclophosphamide) + Surgery + RT	High CD44 correlates with poor clinical response and resistance to chemotherapy
Gvozdenovic et al., 2013 [44]	Osteosarcoma (*n* = 53)	NACT + Surgery	CD44-positive patients had shorter overall mean survival and mean metastasis-free survival
Han et al., 2000 [45]	Leukemia (*n* = 145)	CT (idarubicin, VP-16, Ara-C, or 6-TG)	CD44-high group associated with more frequently expressed in relapsed or refractory cases
Hara et al., 2019 [46]	Esophageal SCC (*n* = 146)	CT (DDP + 5-FU/ACF or DCF) + Surgery	CD44-high group associated with poorer clinical response to treatment
Huh et al., 2014 [47]	Rectal cancer (*n* = 123)	Preoperative CT (5-FU + leucovorin) + Surgery	Elevated pretreatment CD44 predictive of poor tumor regression
Klose et al., 2021 [48]	Rectal cancer (*n* = 218)	Neoadjuvant RCT (5-FU) + Surgery	Presence of CD44 cells associated with impaired overall survival
Koukourakis et al., 2012 [49]	Head and neck SCC (*n* = 74)	CRT (DDP, amifostine, or cetuximab)	High presence of CD44+ cells associated with incomplete response after therapy
Lee et al., 2011 [50]	Breast cancer (*n* = 92)	Primary systemic CT (AD/AC)	CD44+ populations showed higher Ki-67 proliferation index and shorter disease-free survival
Lin et al., 2010 [51]	Head and neck SCC (*n* = 54)	CT/RT	High pretreatment CD44 mRNA levels associated with poor prognosis
Lin et al., 2012 [53]	Breast cancer (*n* = 147)	Surgery + CT	CD44+ phenotype associated with shorter disease-free survival and overall survival
Liu et al., 2012 [52]	Breast cancer (*n* = 135)	NACT + Surgery	High ratio of CD44+ cells less sensitive to chemotherapy
Mikami et al., 2015 [54]	Kidney carcinoma (*n* = 25)	CT (sunitinib)	Patients with CD44-high cells had shorter time to treatment failure and overall survival
Negri et al., 2019 [55]	Colon cancer (*n* = 51)	CT (bevacizumab)	High expression of CD44 predicted reduced progression-free survival and overall survival
Ristamäki et al., 1997 [56]	Lymphoma (*n* = 194)	CRT (bleo-CHOP or M-BACOD) or another anthracycline containing combination)	Patients with high s-CD44 concentrations had poorer survival
Tokunaga et al., 2019 [57]	Breast cancer (*n* = 48)	NACT (anthracycline/taxanes) + Surgery	High pretreatment CD44 expression associated with poor prognosis
Wang et al., 2011 [58]	Gastric carcinoma (*n* = 8)	NACT (DCF + DDP + capecitabine) + Surgery	High CD44 expression associated with worse patient survival
Yamauchi et al., 2018 [59]	Breast Cancer (*n* = 18)	CT (trastuzumab + lapatinib + paclitaxel)	Persistent expression of CD44 associated with poor response to chemotherapy
Saigusa et al., 2012 [60]	Rectal cancer (*n* = 52)	Preoperative CRT (5-FU + UFT) + Surgery	Positive CD44 gene expression is correlated with disease recurrence and poor overall survival
Zhao et al., 2022 [61]	Lung cancer (*n* = 72)	CT (DCF + DDP + capecitabine) ± RT	High baseline HA or CD44 associated with bone metastasis

SCC = squamous cell carcinoma; CRT = chemoradiotherapy; DDP = cisplatin; CT = chemotherapy; NACT = neoadjuvant chemotherapy; 5-FU = 5-fluorouracil; DCF = docetaxel; RT = radiotherapy; VP-16 = etoposide; Ara-C = cytarabine; 6-TG = thioguanine; ACF = adriamycin, cyclophosphamide, ftorafur; UFT = tegafur–uracil; AD = docetaxel + doxorubicin; AC = cyclophosphamide; bleo-CHOP = bleomycin, cyclophosphamide, doxorubicin, vincristine, prednisone; M-BACOD = methotrexate, bleomycin, doxorubicin, cyclophosphamide, vincristine, dexamethasone.

**Table 4:** Number of articles showing the effect of CD44 expression on chemotherapy treatment outcome stratified by different types of intervention (chemotherapy is the first-line treatment all the different types of intervention).

Intervention	Number of Articles Showing the Effect of CD44 Expression Has on Different Types of Intervention		
	Negative Effect	No Effect	Positive Effect
Only Chemotherapy	7 [45,50,51,54,55,59,61]	2 [35,36]	0
Chemotherapy + Radiotherapy	3 [38,49,56]	3 [29,31,34]	2 [23,24]
Chemotherapy + Surgery	10 [39,41,42,44,46,47,52,53,57,58]	5 [25,26,27,30,33]	0
Chemotherapy + Radiotherapy + Surgery	4 [39,43,48,60]	2 [28,37]	1 [22]

**Table 5:** Number of articles showing the effect of CD44 expression on chemotherapy treatment outcome stratified by the most common chemotherapeutic drugs (5-fluorouracil, cisplatin, and docetaxel).

Drug Used	Type of Chemotherapy	Effect of CD44 Expression on Chemotherapy Outcome		
		Negative Effect	No Effect	Positive Effect
5-Fluorouracil	Single	[28,41]	[32,48]	
	Combination	[31,38,43,46,47]	[33,35,37,60]	
Cisplatin	Single	[38]	[29,34]	[23]
	Combination	[38,39,46,49,58,61]	[30,33,34]	
Docetaxel	Single	[46]		
	Combination	[38,50,58,61]	[34]	
Other drugs	Single	[54,55]		[24]
	Combination	[45,46,49,50,56,57,58,60,61]	[30,31,34,35,37]	

**Table 6:** Number of articles showing the effect of CD44 expression on chemotherapy treatment outcome stratified by the types of cancer.

Cancer Type	Number of Articles	Number of Articles Showing Effect of CD44 Expression on Chemotherapy Treatment Outcome		
		Negative	No Effect	Positive Effect
Breast	11	8 [40,43,50,51,52,53,57,59]	3 [25,26,35]	0
Rectal	7	3 [47,48,60]	4 [28,31,32,37]	0
Head and Neck	4	3 [38,49,51]	1 [29]	0
Cervical	3	1 [39]	1 [34]	1 [23]
Esophageal	3	1 [46]	1 [33]	1 [22]
Leukemia	2	1 [45]	1 [36]	0
Osteosarcoma	3	1 [44]	2 [27,30]	0
Colon	2	2 [41,55]	0	0
Kidney	2	2 [42,54]	0	0
Gastric	1	1 [58]	0	0
Glioblastoma	1	0	0	1 [24]
Lung	1	1 [61]	0	0
Lymphoma	1	1 [56]	0	0

## Data Availability

Data is contained within the article and Supplementary Materials.

## Supplementary Materials

The following supporting information can be downloaded at https://www.mdpi.com/article/10.3390/ijms25063141/s1.
