# Supplementary material for: Role of CD44 in Chemotherapy Treatment Outcome: A Scoping Review of Clinical Studies
Source: Int J Mol Sci. 2024 Mar 8;25(6):3141. doi: 10.3390/ijms25063141 (PMC10970610; doi:10.3390/ijms25063141)
Supplement: Supplementary file 1 [file ijms-25-03141-s001.zip › Supplementary Table 1.pdf]

Supplementary Materials

Supplementary Table 1. Kappa scores for title and abstract and full text screening

Title and Abstract Screenings					
**Parts**	**Proportionate Agreement**	**Yes Probability**	**No Probability**	**Random Agreement Probability**	**Cohen's Kappa**
1	0.95	0.02	0.71	0.74	0.81
2	0.92	0.03	0.70	0.73	0.70
3	0.93	0.04	0.64	0.68	0.77
**Full Text Screenings**					
**Parts**	**Proportionate Agreement**	**Yes Probability**	**No Probability**	**Random Agreement Probability**	**Cohen's Kappa**
1	0.96	0.16	0.36	0.52	0.91
2	0.95	0.16	0.37	0.52	0.90
3	0.96	0.03	0.69	0.72	0.87
